# 

**Published:** 1889-12-07

**Authors:** 


### The Student's Column.

### THE VICTORIA UNIVERSITY.

#### DEGREE DAY, NOVEMBER 7, 1889.

The following candidates are qualified for degrees. The names are in alphabetical order in each class and division:

Doctor of Medicine.—John Brown, M.B., Ch.B., Owens; Robert James M'Lean Buchanan, M.B., Ch.B., University; Henry Waytes Pomfret, M.B., Ch.B., Owens.

Bachelor of Medicine and of Surgery.—*First Division.*—Albert Edward Brindley, Owens, distinguished in obstetrics and diseases of women; Theophilus Nicholas Kelynack, Owens, distinguished in medicine, surgery, morbid anatomy, forensic medicine, pharmacology and therapeutics, and anatomy Robert Ellis Lord Owens distinguished in anatomy, materia medica, and pharmacy; James Sinclair M'Gowan, Owens; Arthur James Moss, Owens, distinguished in obstetrics and diseases of women; Frank Lomax Wood, Owens, distinguished in anatomy, materia medica, and pharmacy.

*Second Division.*—Richard Alcock, Owens; Albert Ashton, Owens; John Dunlop, Owens; George Frederick Edwards, Owens, distinguished in obstetrics and diseases of women, and physiology; John William Ellis, University; Charles Frederick Marshall, Owens, distinguished in anatomy; Harry Merrall, Owens; Edward Molyneux, University; Thomas Porter, Owens; James Simcock, Owens; Frederic Wilson Stansfield, Owens; John Clough Thresh, Owens, distinguished in pharmacology and therapeutics, pathology and anatomy; James Wilcock Unsworth, Owens; Arthur Blair Watson Owens; Frederick James Webb, Owens.

### NOTICE TO CORRESPONDENTS.

All MS., Letters, Books for Review, and other matters intended for the Editor, should be addressed The Editor, The Lodge, Porchester Square, London, W.

### Notice to Advertisers and Subscribers.

All Advertisements, Orders for Copies of The Hospital, and Business Communications should be addressed to The Publisher, *not to the Editor*, The Hospital, 139-140, Salisbury-court, Fleet-street, E.C.

### Bound Volumes of "The Hospital."

Vol. VI., containing full page portraits of T.R.H. the Prince and Princess of Wales, and Miss Florence Nightingale, is now ready, 4s., post free. Cases for binding, may be had of the Publisher, at 1s. 6d. each.

### To Residents, Secretaries, and Matrons

The Editor will be glad to have the Regulations and each Report as issued by Asylums, Hospitals, Convalescent and Nursing Institutions, as well as those of other Charities, and an early copy in duplicate of all Appeals and Festival Notices, etc., addressed to *The Editor, The Lodge, Porchester-square, London, W.* Any superintendent, secretary, matron, or other chief official who regularly sends such information may be registered, on application to the Publisher, to receive free of cost a cover for binding The Hospital in half-yearly volumes.

### Scraps and Gleanings.

Melton Parish Church has sent £32 to Leicester Infirmary.

At Helsingfors there are 133 medical students, of whom only two are women.

Dr. Wheeler has been appointed resident surgeon to Belfast Royal Hospital.

Mr. A. W. W. Lee has been appointed house surgeon to Manchester Infirmary.

A pension for incurables is to be founded in memory of the late Dr. Radcliffe.

The Evelina Hospital has issued a very pretty little pocket almanac and diary for 1890.

The Royal Albert Edward Infirmary, Wigan, is to have a new day room for male patients.

During October 14,756lb. of diseased or unsound meat was seized in Edinburgh by the authorities.

Mr. George F. Blake has been appointed registrar to the Royal College of Surgeons in Ireland.

The lowest death rate in Ireland last month was at Dundalk and Kilkenny, the highest at Limerick and Belfast.

The Hereford Eye and Ear Hospital supplies no religious service to its patients; the fact has been brought forward in the local press.

The Spanish Medico-Chirurgical Society offers two prizes of £10 each for two essays on given subjects. The essays may be written in English.

Sir Richard Webster, Q.C., M.P., Attorney-General, has accepted the office of a vice-president to the Dental Hospital of London, Leicester-square.

The "Charities Register and Digest" is the title of an elaborate work just published by Longmans and the Charity Organisation Society.

The Rev. Hugh B. Chapman will preach at Westminster Abbey on Monday afternoon, Dec. 23rd, on "The Life and Death of Father Damien."

The Kensington Home for Crippled Boys is asking healthy children throughout the land to make a collection of pence for them on Christmas morning.

The Lord Mayor has consented to preside at a festival dinner in aid of the Hampstead Home Hospital, at the Hotel Metropole on Thursday, the 20th March next.

A subscription dinner, in aid of the Leprosy (Father Damien) Fund is fixed for the 13th of January, at the Hotel Metropole. The Prince of Wales will occupy the chair as president.

The value has been sworn at £15,302 3s. 4d. of the personal estate of the late Sir William Tindal Robertson, M.D., M.P., of 9, Belgrave-terrace, Brighton, who died on the 12th ult.

At Bradford a meeting of ministers and clergymen has been held to organise a course of action for increasing the contributions from places of worship to the Bradford Joint Hospital Fund.

Lord Windsor has told the Rev. W. Sweet Escott, rector of Penarth, that in addition to a contribution of £3,000 towards the building fund he will give a site for the new church on the Victoria-road.

Dr. Robson Roose, who recently enquired into the condition of the lepers in Norway, for whose amelioration there are established several large hospitals, is preparing an elaborate report on his mission.

The twenty-second annual Hospital Sunday at Richmond was held on the 10th November, and the receipts to the present time are £486 16s. 6d., being over £115 in excess of last year's collections.

The Wesleyans have received a windfall in the shape of some £10,000 from the executors of the late Sir William McArthur, which will be applied to the purposes of the extension of Methodism in Great Britain.

St. Petersburg is suffering from a very unpleasant visitation—an epidemic of influenza, which, instead of decreasing, appears to be doing the reverse. It is supposed that a fourth or fifth of the population has been attacked.

Dr. A. Sheen, of Cardiff, criticises Dr. Rentoul's scheme for the reform of medical charities. Dr. Sheen says a well-managed infirmary and provident dispensary, working in harmony, should meet all the necessities of the sick poor.

The wards for internal diseases at the new Vienna hospital (Franz-Josephs-Spital) have now been opened. The hospital, which is some distance outside the town, is built on the pavilion system, and when completed will afford accommodation for 600 patients.

In Germany and in Russia there are special hospitals for the treatment of morphinomaniacs. It is affirmed that in one of the countries they have their "morphine parties," just as we have in England our tea or wine parties, and that the company innoculate each other with the drug.

A dreadful occurrence is reported from Blackfoot, Idaho. A large insane asylum there caught fire, and although the most strenuous efforts were made to rescue the unfortunate inmates, eight perished before they could be brought to a place of safety. The building was burned out, and the loss is estimated at £60,000.

At a meeting of the Metropolitan Asylums Board last Saturday, it was reported that for the two weeks ended Thursday last there were 1,829 fever patients, mostly scarlet fever, in the hospitals under the board, being an increase of 86 over the number for the two preceding weeks. There was only one smallpox patient.

T. H. Arnold Chaplain, M.B.Cantab., &c., has been appointed resident medical officer of the City of London Hospital for Diseases of the Chest, Victoria Park, in succession to Dr. Raymond Courteen resigned. Robert Lachlan Pinkerton, M.B., C.M. Glas., has been appointed house physician for six months, commencing 1st January, 1890.

At Wloclawek, in Poland, a man named Pawlikowski has just died at the age of 115. He fought through Kosciuszko's wars and through Napoleon I.'s Russian campaign. He was working in the fields up to last year. His father is said to have lived to the age of 126, and one of his brothers died at 116. He leaves three sisters aged 102, 99, and 93 respectively.

